# Concurrent Norovirus Outbreaks Associated with Consumption of Oysters Harvested in Mexico — California, December 2023–January 2024

**DOI:** 10.15585/mmwr.mm7413a2

**Published:** 2025-04-17

**Authors:** Sophie Zhu, Christina Grant, Chao-Yang Pan, Brandon Adcock, Annie Kao, Sarah Stous, Lisa Yee, Olivia Springfield, Madeline Poranski, Audrey Kennar, Mark Beatty, Seema Shah, Heather Watson, Heather Buonomo, Anna Liza M. Manlutac, Heriberto Lima, Devon Mendez, Bianca Clark, Marifi Pulido, Melina Bakshi, Charlene Contreras, Nicole Green, Taylor Burleson, Jenafer Forester, Stephen W. Klish, Matthew Feaster, Elizabeth V. Taylor, Nora Balanji, Vanna Kho, April Hatada, Chelsea Wright, Christina Morales, Melissa Abbott, Floyd Raymond Burditt, Elisa Elliot, Jessica L. Jones, Marshall Kinsey, Michael Lombardi, Kristina Phelps, Jaquelina W. Woods, Akiko Kimura, Katherine Lamba

**Affiliations:** ^1^California Department of Public Health; ^2^Epidemic Intelligence Service, CDC; ^3^County of San Diego Healthy and Human Services Agency, San Diego, California; ^4^Los Angeles County Department of Public Health, Los Angeles, California; ^5^County of Orange Health Care Agency Public Health Services, Santa Ana, California; ^6^City of Pasadena Department of Public Health, Pasadena, California; ^7^Long Beach Department of Health and Human Services, Long Beach, California; ^8^Human Foods Program, Food and Drug Administration, College Park, Maryland.

**Summary**

**What is already known about this topic?**

Consumption of contaminated raw oysters is a common cause of foodborne illness outbreaks.

**What is added by this report?**

During December 2023–January 2024, approximately 400 persons across eight California local health jurisdictions reported gastrointestinal illness after consumption of raw oysters. The investigation identified two concurrent but unrelated outbreaks attributable to norovirus and other viral enteric pathogens. In the second outbreak, oysters might have been contaminated during wet storage of live oysters at a location separate from the original growing area.

**What are the implications for public health practice?**

Raw oysters are a continuing source of enteric illness. Producers and distributors should be aware of and prevent shellfish contamination in wet storage. Consumers should cook oysters to 145°F (62.8°C) before consumption. Concurrent outbreaks of foodborne illness with similar modes of transmission can be unrelated and should be confirmed by product traceback.

## Abstract

Norovirus is the most common cause of foodborne illness outbreaks in the United States. In January 2024, local health jurisdictions and the California Department of Public Health (CDPH) identified two concurrent norovirus outbreaks across eight Southern California local health jurisdictions. CDPH was notified in late December 2023 and early January 2024 of gastrointestinal illnesses in persons who consumed raw oysters from food service facilities in San Diego County (outbreak 1). Additional illness reports came from multiple jurisdictions that included Los Angeles County and other areas in Southern California (outbreak 2). In total, approximately 400 persons across eight local health jurisdictions reported gastrointestinal illness after raw oyster consumption. A multiagency investigation confirmed that outbreaks 1 and 2 were unrelated, and implicated oysters were traced to two separate, nonoverlapping harvest regions in Mexico. A total of 179 outbreak-associated cases, including 24 laboratory-confirmed norovirus cases, were identified. Patient samples from both outbreaks identified norovirus genogroups I and II; other enteric viruses (sapovirus, astrovirus, rotavirus, and adenovirus) were also identified from one or both outbreaks. Noroviruses were genetically related by genotype within each outbreak but dissimilar between outbreaks. In outbreak 2, oysters might have been contaminated at a location separate from the original growing area, also known as wet storage. Concurrent outbreaks with similar modes of transmission can be unrelated, and the source for each should be confirmed through traceback. Proper storage and handling of shellfish is essential to maintaining safety of food products to consumers. Cooking oysters to 145°F (62.8°C) is recommended before consumption.

## Investigations and Findings

### Epidemiologic Investigation

**Illness reports.** On December 31, 2023, a food service facility notified the California Department of Public Health (CDPH) and County of San Diego (CoSD) Health and Human Services Agency (HHSA) of gastrointestinal (GI) illness reports among 37 persons who had consumed oysters there during December 27–29, 2023; these illnesses were confirmed by CoSD public health laboratory on January 2, 2024, as caused by norovirus infections. By January 9, CoSD HHSA identified 16 additional GI illness complaints from customers who consumed oysters at three locations of a local restaurant chain unrelated to the first facility. 

On January 12, 2024, CDPH received reports from Southern California local health jurisdictions within and surrounding Los Angeles County of GI illness and norovirus infections among persons who consumed oysters at additional food service facilities. By January 16, 140 illness reports were associated with 51 facilities throughout greater Los Angeles and San Diego (Figure).

**FIGURE:**
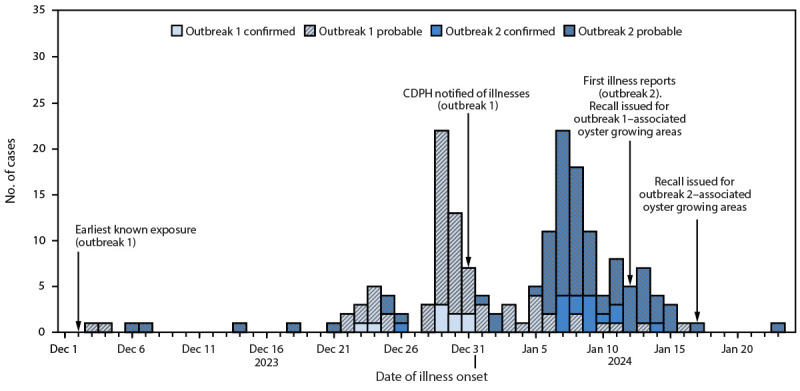
Illness onset dates of confirmed* and probable^†^ norovirus cases in two outbreaks associated with consumption of raw oysters harvested in Mexico — California, December 2023–January 2024 **Abbreviation:** CDPH = California Department of Public Health. * Laboratory-confirmed norovirus infection with illness onset during December 1, 2023–January 31, 2024, in a person who consumed oysters in Southern California 12–48 hours before illness onset. ^†^ Vomiting or diarrhea with illness onset during December 1, 2023–January 31, 2024, in a person who consumed oysters in Southern California 12–48 hours before illness onset.

These GI illnesses were initially considered a single oyster-associated norovirus outbreak because of similarities in the temporal and geographic patterns of cases. Norovirus cases are not reportable in California; however, all outbreaks associated with norovirus are reportable by health care providers and local health jurisdictions and are investigated by public health agencies when possible. CDPH, in collaboration with local health jurisdictions, established a case definition; compiled clinical, exposure, and laboratory information; and performed traceback for implicated oysters. The investigation objectives were to determine the outbreak scope and oyster source, halt further transmission, and identify preventative measures. This activity was reviewed by CDPH and CDC, deemed not research, and was conducted consistent with applicable federal law, state law, and CDC policy.*

**Case definition.** A confirmed outbreak-associated case was defined as a laboratory-confirmed norovirus infection in a person with illness onset during December 1, 2023–January 31, 2024, who consumed oysters in Southern California 12–48 hours before illness onset. A probable case was occurrence of vomiting or diarrhea (three or more loose stools in a 24-hour period) with illness onset during December 1, 2023–January 31, 2024, in a person who consumed oysters in Southern California 12–48 hours before illness onset. On January 17, 2024, Southern California local health jurisdictions deployed an electronic survey to persons with potential cases who reported their illness to public health agencies and followed up with telephone interviews to determine if the outbreak case definition had been met and to collect additional clinical and oyster exposure information. Excluding the initial ill persons from San Diego who reported their illnesses to the food service facility, all cases were passively reported by ill persons to public health agencies after release of health advisories and recall notices. 

**Patient characteristics.** Based on information provided by local health departments, approximately 400 persons from eight local health jurisdictions reported illness after consuming oysters; among those interviewed by telephone, 179 persons met the confirmed or probable case definition for illness (Table 1). Patients were median age 40 (IQR = 32.3–55) years, and 100 (56%) were female. Outbreak 1 cases included patients who ate at four facilities exclusively in San Diego County; outbreak 2 cases included patients who ate at 60 facilities in Los Angeles County and surrounding local health jurisdictions. Oysters were consumed during December 2, 2023–January 19, 2024, by 78 outbreak 1 patients and 101 outbreak 2 patients. Illness onset for the two outbreaks occurred during December 3, 2023–January 21, 2024. The median duration of signs and symptoms (e.g., vomiting, diarrhea, nausea, stomach pain or cramps, and fever) was 72 (IQR = 36–120) hours. Twenty-one (12%) patients sought medical care, two (1%) were hospitalized, and no deaths were reported.

**TABLE 1:** Characteristics of persons with probable and confirmed norovirus illnesses associated with consumption of raw oysters from Mexico — California, December 2023–January 2024

Characteristic	No. (%)		
	Total	Outbreak 1	Outbreak 2
**Total no. of cases**	**179**	**78**	**101**
Probable	**78 (43.6)**	69 (88.5)	86 (85.1)
Confirmed	**101 (56.4)**	9 (11.5)	15 (14.9)
**Median age, yrs (IQR)**	**40 (32.3–55.0)**	42 (32.3–63)	38.5 (32.8–49)
**Sex**			
Female	**100 (55.9)**	40 (51.3)	60 (59.4)
Male	**78 (43.6)**	38 (48.7)	40 (39.6)
Unknown	**1 (0.5)**	0 (—)	1 (1.0)
**Consumed raw oysters**	**179 (100.0)**	78 (100.0)	101 (100.0)
**Symptom duration, hrs, median (IQR)**	**72 (36.0–120.0)**	96 (48.0–124.0)	48 (27.0–93.5)
**Vomiting**	**145 (81.0)**	63 (80.8)	82 (81.2)
**Diarrhea**	**160 (89.4)**	76 (97.4)	84 (83.2)
**Nausea**			
Yes	**72 (40.2)**	0 (—)	72 (71.3)
No	**7 (3.9)**	0 (—)	7 (6.9)
Unknown	**100 (55.9)**	78 (100.0)	22 (21.8)
**Stomach pain or cramps**			
Yes	**146 (81.6)**	60 (76.9)	86 (85.1)
No	**31 (17.3)**	16 (20.5)	15 (14.9)
Unknown	**2 (1.1)**	2 (2.6)	0 (—)
**Fever**			
Yes	**84 (46.9)**	45 (57.7)	39 (38.6)
No	**86 (48.0)**	30 (38.5)	56 (55.4)
Unknown	**9 (5.1)**	3 (3.8)	6 (6.0)
**Sought any medical care (outpatient, emergency department, urgent care, or telehealth)**			
Yes	**21 (11.7)**	10 (12.8)	11 (10.9)
No	**15 (8.4)**	0 (—)	15 (14.9)
Unknown	**143 (79.9)**	68 (87.2)	75 (74.2)
**Hospitalized**	**2 (1.1)**	0 (—)	2 (2.0)

### Environmental Health Investigation

CDPH and environmental health staff members from local health jurisdictions completed concurrent traceback investigations and collected shellfish tags (federally required records attached to raw molluscan shellfish that must contain harvest, dealer, and certification information as well as consumption warnings) and invoices at all facilities associated with illness reports. The traceback investigation identified that the two outbreaks were unrelated.

**Sources of outbreak-associated oysters.** A common distributor, distributor A, was identified for the oysters associated with outbreak 1. These oysters were harvested on December 18 and December 27, 2023, from the growing region of Bahia Salina Sonora, Mexico. The harvest area, which might have had fecal contamination from sewage runoff, was the suspected contamination source for outbreak 1 based on investigations by Mexican authorities. Distribution of these oysters was limited to facilities in San Diego County.

Oysters associated with outbreak 2 were distributed to facilities throughout Los Angeles and Southern California, including facilities in San Diego County. Oysters associated with outbreak 2 were harvested during November 21–December 29, 2023, from the Laguna de Guerrero Negro and Laguna Manuela growing areas of Baja California, Mexico. These harvest areas were in a similar region but located approximately 25 miles apart; however, all outbreak 2–associated oysters had been held in wet storage during November 22, 2023–January 5, 2024, in Rincón de Ballenas, a natural body of water located in the Ensenada Bay in Baja California, Mexico. Wet storage is the practice of storing live, market-ready oysters in natural bodies of water or in tanks containing seawater at a location that can be separate from the original growing area and can contain multiple lots from different harvest areas.

Environmental traceback confirmed the discrete nature of outbreaks 1 and 2, with no identified shared wet storage or harvest areas between the two outbreaks, and different oyster types associated with each. During inspection of implicated facilities in Southern California, multiple gaps in food safety procedures were identified. This included mislabeling of shellfish tags; missing information regarding harvest dates, wet storage dates, and harvest locations; and identification of dealers who were distributing oysters without certifications. CDPH issued notices of violations to inspected facilities that did not adhere to regulatory guidelines.^†^

### Laboratory Investigation

Twelve patients from outbreak 1 and 17 from outbreak 2 submitted stool specimens within 10 days of symptom onset to a local public health laboratory to be tested for enteric viral pathogens. CDPH used reverse transcription real-time polymerase chain reaction (RT-qPCR) to confirm local laboratory results and to screen stool samples for viruses following the national norovirus outbreak surveillance network (CaliciNet) protocol (*1*). Conventional RT-PCR, targeting capsid and polymerase genes, was performed, amplicons were genotyped by Sanger sequencing, and results were submitted to the CaliciNet national database. Rotavirus typing was performed at CDC. Among the 29 specimens tested, 27 were positive for at least one enteric virus (Table 2). Patient samples from outbreak 1 yielded norovirus genogroup I (eight specimens), II (seven), sapovirus (six), human astrovirus (three), and rotavirus (two). Patient samples from outbreak 2 yielded norovirus genogroup I (three), II (15), sapovirus (five), rotavirus (two), and adenovirus (one). Among all patients with positive specimens, one to five enteric viruses were detected per specimen. Viruses were genetically similar by norovirus genotype (i.e., the same genotype with some nucleotide differences) and other enteric virus strains within each outbreak but isolates in the two outbreaks were dissimilar. Oysters collected from a distributor and from the same harvest area as those in the implicated outbreak 2 lots were tested by the Food and Drug Administration (FDA) using RT-qPCR for viral pathogens; no viral pathogens were detected.^§^

**TABLE 2:** Laboratory sequencing results of specimens from confirmed norovirus cases* associated with raw oyster consumption from Mexico — California, December 2023–January 2024

Case ID	Outbreak	Local health jurisdiction	Facility	Norovirus genogroup	Other viruses
1	1	San Diego	A	I	Sapovirus GIV.1
2	1	San Diego	A	I/II	—
3^†^	1	San Diego	A	—	Sapovirus GII.2
4	1	San Diego	A	II	Astrovirus 4
5^†^	1	San Diego	A	—	Sapovirus GII.5
6	1	San Diego	A	I	—
7	1	San Diego	B	I/II	Rotavirus G3P[8], Sapovirus GI.3, Astrovirus 4
8	1	San Diego	B	I/II	Rotavirus, Astrovirus 5
9	1	San Diego	B	I/II	Sapovirus GIV.1
10	1	San Diego	B	I/II	—
11	1	San Diego	B	I/II	Sapovirus GIV.1
12	2	San Diego	C	I/II	—
13	2	San Diego	C	II	Sapovirus GIV.1
14	2	San Diego	D	II	—
15	2	San Diego	D	II	—
16	2	San Diego	E	II	Sapovirus GIV.1
17	2	San Diego	E	II	—
18	2	San Diego	F	I/II	—
19	2	San Diego	G	II	Rotavirus G3P[8], Sapovirus GIV.1, Adenovirus 31
20^†^	2	San Diego	G	—	Sapovirus GIV.1
21	2	San Diego	H	II	Rotavirus G3P[8]
22	2	San Diego	H	I/II	Sapovirus GI.1
23	2	San Diego	I	II	—
24	2	Los Angeles	J	II	—
25	2	Los Angeles	K	II	—
26	2	Pasadena	L	II	—
27	2	Orange	M	II	—

**Abbreviation**: ID = identification.

* Two cases had specimens that tested negative for enteric viruses.

^†^ These persons received negative norovirus test results but received positive test results for the other enteric viruses listed in the table.

## Public Health Response

In late December 2023, FDA released a health advisory for contaminated oysters from certain harvest areas in Bahia Salina, Sonora, Mexico in response to outbreak 1 (*2*). On January 12, 2024, distributor A issued a recall. On January 17, 2024, in response to outbreak 2, FDA issued a second health advisory for contaminated oysters from some harvest areas in Baja California, Mexico, leading to recall of the product (*3*). Growing areas were closed by Mexican authorities for 21 days after confirmation of positive norovirus samples from patients with outbreak-associated cases. CDPH and California local health jurisdictions issued advisories and press releases to consumers, restaurants, and retailers not to eat, serve, or sell any oysters that were grown in certain regions of Mexico implicated in the investigation (*4*–*8*).

## Discussion

Geographic separation of the implicated harvest locations, different distributors, and distinct virus genotypes and oyster types confirmed the occurrence of two concurrent but separate norovirus outbreaks associated with consumption of raw oysters from Mexico. The epidemiologic and laboratory investigation identified raw oysters from two sources in Mexico as the likely source of both norovirus outbreaks and other enteric virus detections. Continuing multiple traceback efforts even after identifying the source of outbreak 1 oysters led to recognition that the two outbreaks were unrelated.

Multiple foodborne illness outbreaks have been associated with consuming raw oysters, including a multistate outbreak of norovirus illness in the Southern United States (*9*). Early communication among CDPH, local health jurisdictions, federal partners, and facilities permitted rapid identification and response to illness reports as well as traceback and source determination.

Oysters can become contaminated with enteric viruses from human fecal matter in their growing environment during transport or wet storage, and during handling at food service processing facilities before human consumption. The recognition of wet storage as the likely source of contamination in outbreak 2 was the result of the growing area investigation by Mexican authorities. Although some oysters could have entered wet storage already contaminated, the geographic separation of harvest areas (approximately 25 miles) in outbreak 2 points to wet storage playing a role. Wet storage, both in natural bodies of water and land-based systems, is a common practice globally that prolongs the time from harvest to distribution and consumption. Wet storage was previously identified as a source of contamination for bacterial outbreaks in raw oysters in Hong Kong. However, no documented enteric viral outbreaks have previously been associated with wet storage (*10*).

### Limitations

The findings in this report are subject to at least three limitations. First, the source of contamination of outbreak 1 was suspected but not definitively confirmed as the harvest area. Second, because of the role of wet storage in outbreak 2, the definitive source of contamination could not be confirmed. Finally, passive reporting might have led to underascertainment of cases. 

### Implications for Public Health Practice

Although the exact source of contamination for outbreak 2 was not determined, shellfish growers should consider measures to prevent possible contamination of oysters from multiple harvest lots during wet storage.^¶^ Producers should be aware of the potential risks associated with wet storage of oysters and should take steps to reduce exposure risks to consumers. Consumers should be aware of the health risks for raw oyster consumption, which can be avoided through cooking; the recommended method of oyster preparation is to thoroughly cook raw oysters to 145°F (62.8°C) before consumption.**

## Footnotes

* 45 C.F.R. part 46, 21 C.F.R. part 56; 42 U.S.C. Sect. 241(d); 5 U.S.C. Sect. 552a; 44 U.S.C. Sect. 3501 et seq.

† https://www.fda.gov/media/143238/download?attachment

§ https://www.fda.gov/food/laboratory-methods-food/bam-chapter-26-and-appendices-concentration-extraction-and-detection-enteric-viruses-food

¶
https://www.fda.gov/media/181370/download?attachment

**
https://www.foodsafety.gov/blog/safe-selection-and-handling-fish-and-shellfish
